# Dissociable diffusion MRI patterns of white matter microstructure and connectivity in Alzheimer’s disease spectrum

**DOI:** 10.1038/srep45131

**Published:** 2017-03-24

**Authors:** Nhat Trung Doan, Andreas Engvig, Karin Persson, Dag Alnæs, Tobias Kaufmann, Jaroslav Rokicki, Aldo Córdova-Palomera, Torgeir Moberget, Anne Brækhus, Maria Lage Barca, Knut Engedal, Ole A. Andreassen, Geir Selbæk, Lars T. Westlye

**Affiliations:** 1 NORMENT, KG Jebsen Centre for Psychosis Research, Division of Mental Health and Addiction, Oslo University Hospital & Institute of Clinical Medicine, University of Oslo, Norway; 2 Department of Medicine, Diakonhjemmet hospital, Oslo, Norway; 3 Norwegian National Advisory Unit on Ageing and Health, Vestfold Hospital Trust, Tønsberg, Norway; 4 Department of Geriatric Medicine, The Memory Clinic, Oslo University Hospital, Oslo, Norway; 5 Department of Psychology, University of Oslo, Oslo, Norway; 6 Centre for Old Age Psychiatric Research, Innlandet Hospital Trust, Ottestad, Norway

## Abstract

Recent efforts using diffusion tensor imaging (DTI) have documented white matter (WM) alterations in Alzheimer’s disease (AD). The full potential of whole-brain DTI, however, has not been fully exploited as studies have focused on individual microstructural indices independently. In patients with AD (n = 79), mild (MCI, n = 55) and subjective (SCI, n = 30) cognitive impairment, we applied linked independent component analysis (LICA) to model inter-subject variability across five complementary DTI measures (fractional anisotropy (FA), axial/radial/mean diffusivity, diffusion tensor mode), two crossing fiber measures estimated using a multi-compartment crossing-fiber model reflecting the volume fraction of the dominant (f1) and non-dominant (f2) diffusion orientation, and finally, connectivity density obtained from full-brain probabilistic tractography. The LICA component explaining the largest data variance was highly sensitive to disease severity (AD < MCI < SCI) and revealed widespread coordinated decreases in FA and f1 with increases in all diffusivity measures in AD. Additionally, it reflected regional coordinated decreases and increases in f2, mode and connectivity density, implicating bidirectional alterations of crossing fibers in the fornix, uncinate fasciculi, corpus callosum and major sensorimotor pathways. LICA yielded improved diagnostic classification performance compared to univariate region-of-interest features. Our results document coordinated WM microstructural and connectivity alterations in line with disease severity across the AD continuum.

Alzheimer’s disease (AD) is the leading cause of dementia, a devastating group of brain disorders affecting 47 million individuals globally^1^. The insidious AD neuropathological process begins years before dementia onset^2^, and is preceded by mild cognitive impairment^3^. Since disease-modifying treatments for established AD dementia have failed^4^, the field has shifted focus towards earlier intervention with some promise^5^. It is currently a key priority in AD research to develop neuroimaging methods that can accurately identify individuals in the earliest clinical stages of the disease, such as the stages of subjective (SCI) or mild cognitive impairment (MCI), for targeting early intervention and stratifying groups in clinical trials^6^.

Although historically primarily associated with regional gray matter (GM) loss, including medial temporal lobe atrophy (MTA), converging lines of evidence point to a direct role of white matter (WM) degeneration in AD pathogenesis^7^,^8^. Supporting a critical role of WM microstructure in AD, the apolipoprotein E (APOE) gene, of which the ε4 allele polymorphism is the strongest known genetic risk factor for sporadic AD^9^, codes for a cholesterol transporter directly involved in WM myelination^10^. Diffusion weighted magnetic resonance imaging (diffusion MRI) is sensitive to a range of WM characteristics, including myelin-related processes^11^, and WM diffusion tensor imaging (DTI) alterations have been included among the phenotypic signatures of APOE in non-pathological and preclinical stages^12^,^13^,^14^,^15^. Based on a multiparametric imaging study of a transgenic mouse model of tauopathy, which is a hallmark of AD development, it was concluded that DTI is highly sensitive to processes preceding detectable structural and neurodegenerative processes^16^. Supporting DTI as a sensitive index of microstructural WM degeneration in AD^8^,^17^, meta-analyses revealed converging evidence of increased mean diffusivity (MD) and decreased fractional anisotropy (FA) with increased disease severity^18^, with comparable effect sizes for DTI and MTA for distinguishing AD and MCI patients from healthy controls^19^. These findings suggest that DTI may be complementary to, and as sensitive as the canonical MTA pattern^20^.

Still, only a few studies have tested the accuracy of diffusion MRI metrics in classifying between AD, MCI and healthy controls^21^,^22^. Furthermore, whereas FA and MD are the most studied DTI indices, a range of alternative measures can be calculated, including other voxel-wise characteristics of the diffusion tensor, microstructural indices based on multi-compartment models (e.g., neurite orientation dispersion and density imaging (NODDI^23^) and restriction spectrum imaging (RSI^24^), and tractography based connectivity measures. Integrated considerations of such measures provide a comprehensive description of the WM microstructural anatomy, and may yield complementary information about WM involvement in AD. For example, in an elegant and powerful study using decomposition of intra-voxel fiber orientations and probabilistic tractography, areas of crossing WM fibers were particularly vulnerable to AD, where regionally increased mode of anisotropy (MO) in AD was interpreted to reflect degree of crossing fibers and differential sparing of sensorimotor tracts^25^.

While diffusion MRI data can be used to generate a rich set of WM indices, studies combining complementary DTI measures in multivariate data fusion analysis of AD classification are lacking. Instead, most studies to date have used one or several regions of interest (ROI) or voxel-based approaches assessing one or more DTI indices in separate analyses (see ref. 26 for an exception). Whereas considering different diffusion MRI metrics in independent tests may increase sensitivity compared to only considering one modality^27^, univariate analysis of multiple imaging indices may both be cumbersome and involve sacrificing discriminatory power due to poor modeling of shared variance between measures^28^.

In an attempt to overcome these limitations, we perform a comprehensive analysis fusing eight DTI based measures obtained from 164 subjects across a clinical AD continuum by means of linked independent component analysis (LICA)^28^,^29^. LICA provides an effective way to downsample data with a vast number of voxels to a manageable number of biologically interpretable features.

The main goals were to assess the LICA features to gain new knowledge about WM patterns involved in AD and its tentative clinical precursors and to evaluate the diagnostic utility of these patterns compared to traditional approaches. Specifically, we hypothesized increased diagnostic sensitivity by efficiently modeling shared variance between complementary diffusion MRI measures^28^. Given the hypothesized modulating role of APOE genotype status on WM microstructure and connectivity^13^, we tested for differences between carriers and non-carriers of the APOE ε4 risk allele within and across groups, and also tested if carriers would show more AD-like WM characteristics by comparing the AD classifier’s decision probability between carriers and non-carriers in the SCI and MCI groups.

To this end, we compared our LICA results with univariate voxel-based analyses on the same dataset. We used machine learning to perform pairwise group classification to estimate the diagnostic accuracy at the individual level. We performed the same classification procedure using single metrics alone, and with the LICA features added, and compared performance across approaches. We report sensitivity and specificity for the feature showing the best performance overall to compare with current, established biomarkers of AD^30^.

## Results

Main effects of diagnosis, age, sex and head coil type on subject loadings from each LICA component are presented in Supplementary Fig. S1, and results for pairwise group comparison in terms of Cohen’s *d* are detailed in Supplementary Fig. S2. Sample characteristics are detailed in Table 1.

### One component reflects co-variance patterns of all diffusion MRI measures and is highly sensitive to diagnosis

**Figure 1:**
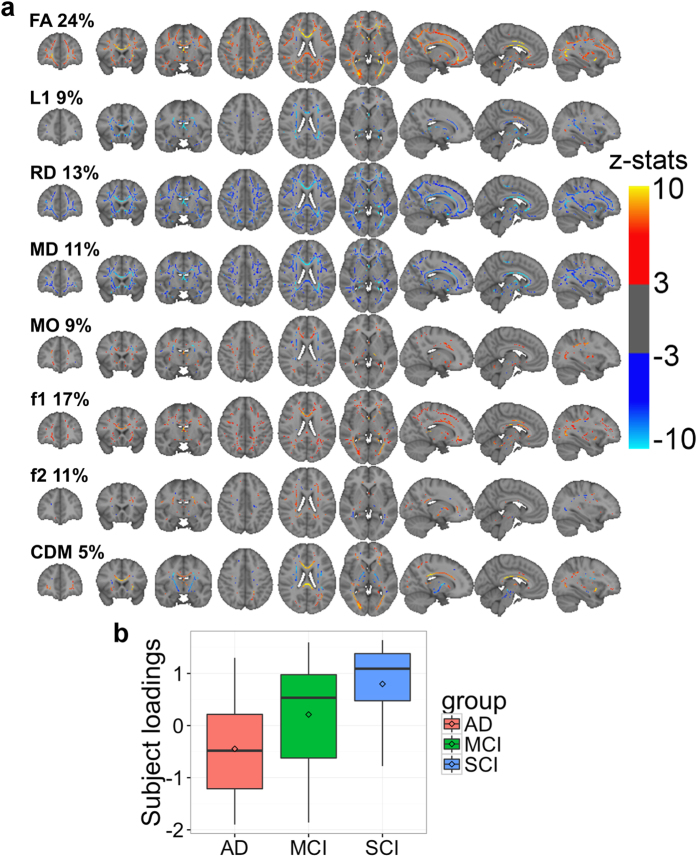
Spatial maps (**a**) and subject loadings distribution (**b**) of IC0. The spatial maps represent the thresholded z-scores (3 < |z| < 10). In the spatial maps, the weights (in percentage) indicate the relative contribution of each measure to the component at the group level. In the subject loading plot, the box represents the 25% and 75% quantiles, the horizontal bar in the box representing the median, and the diamond the mean. The plot shows a graded pattern of AD < MCI < SCI.

Independent component #0 (IC0) explained 45.6% amount of the total variance in the data (Supplementary Fig. S3), captured common variance across all of the eight diffusion MRI measures (Fig. 1) and showed a highly significant effect of diagnosis (corrected for multiple comparisons, *F*_2,158_ = 16.5, *p* < 0.001, Supplementary Fig. S1). Note that although we used a model order of 25 for the present study, post-hoc analyses showed that the main effect of diagnosis for the primary component (IC0) remained stable across a broad range of model orders (Supplementary Fig. S4).

The AD group showed weaker subject loadings than both MCI and SCI (AD – MCI: Cohen’s *d* = −0.58, *p* = 0.03, AD – SCI: *d* = −0.88, *p* = 1.0e-5). Age was strongly negatively associated with IC0 (*F*_1,158_ = 60.3, *p* < 0.001, Supplementary Fig. S1, Supplementary Fig. S14), whereas there were no associations with head coil (*F*_1,158_ = 0.59, *p* = 1, Supplementary Fig. S1).

As indicated by Fig. 1b, weaker subject loadings on IC0 in the AD group reflected widespread correlated decreases in anisotropy (FA, MO) along with increases in diffusivity (L1, RD, MD), encompassing the occipital, temporal, parietal and frontal WM as well as the corpus callosum and fornix. Localized increased anisotropy in AD (both FA and MO) was also seen, essentially restricted to the centrum semiovale. Also, the localized regions of relative increases in FA, MO and CDM in AD overlapped with projection fiber pathways, including the corticospinal tract and corona radiata bilaterally.

The dominant fiber map (f1) of IC0 resembled the FA map indicating preservation of the principal fibers in individuals without dementia (SCI > MCI > AD in subject loadings, Fig. 1b) in most WM regions, with stronger positive weighting seen in the fornix and genu of the corpus callosum. Compared to MCI and SCI, the AD group also showed increased f1 in small regions, mostly implicating the corticospinal tract and centrum semiovale. Increased f2 was observed in most of the centrum semiovale and splenium of the corpus callosum, indicating preservation of the secondary fiber direction in patients without dementia (SCI > MCI > AD, Fig. 1b). The f2 map also showed small and scattered areas of negative weightings, including the bilateral posterior thalamic radiation and the optic radiation, indicating preserved non-dominant fiber direction in AD patients.

The CDM map revealed widespread group differences in connection density (SCI > MCI > AD) of the entire corpus callosum and in several association tracts, including the fornix and the bilateral uncinate. In addition, this map also revealed decreased CDM, essentially implicating the corticospinal tracts bilaterally, suggesting relative increased connection density in AD patients in these tracts.

### Associations between other LICA components and diagnosis

We found a significant main effect of diagnosis on a second independent component, IC21 (*F*_2,158_ = 9.19, *p* = 0.0042, corrected), which explained 0.97% of total variance in the data (Supplementary Fig. S3). The component involved mainly CDM implicating anterior corpus callosal and internal capsule fibers bilaterally (Supplementary Fig. S5). Post-hoc tests revealed significantly higher IC21 subject loadings in AD compared to MCI (AD – MCI: Cohen’s *d* = 0.68, *p* = 0.002), suggesting increased CDM in these tracts in AD.

### Relationships between LICA components and cognition

We compared the two independent components that showed a main effect of diagnosis with MMSE. IC0 showed a positive association with MMSE across groups (Cohen’s *d* = 0.74, *p* < 0.001), accounting for age, sex and head coil. However, no significant association was observed within groups (*p* > 0.1). Similarly, IC21 showed a negative association with MMSE across groups (*d* = −0.61, *p* < 0.001). Within group analyses showed a negative association within AD (*d* = −0.53, *p* = 0.026, uncorrected) and no association within MCI or SCI (*p* > 0.1). We also performed exploratory analyses of subject loadings and episodic memory, processing speed and executive function (Supplementary Table S1) for a subset of patients for whom the data were available. Here, executive function as indexed by the Trail making test (type B) was inversely related to the subject loadings of IC0 for the whole sample (*d* = −0.44, *p* = 0.037, accounting for age, sex and head coil), but failed to reach statistical significance within each diagnosis group (*p* > 0.1).

### Group comparisons using univariate voxel-wise diffusion MRI analyses

Supplemental Fig. S6 shows results from the univariate permutation testing of each of the eight diffusion MRI measures separately. Briefly, the analyses revealed significant (*p* < 0.05, corrected for multiple comparisons across space) group differences between SCI and AD in several metrics, reflecting decreased FA, f1, f2 and MO and increased RD, L1, MO and CDM in AD. Further, we found a small region at the left superior corona radiata with increased MO in AD compared to MCI, but no significant differences between SCI and MCI. Histograms of effect sizes obtained from the univariate TBSS analyses as well as GLM on the LICA features are shown in Supplementary Fig. S7.

### Classification of diagnostic groups using LICA and conventional ROI diffusion MRI features

**Figure 2:**
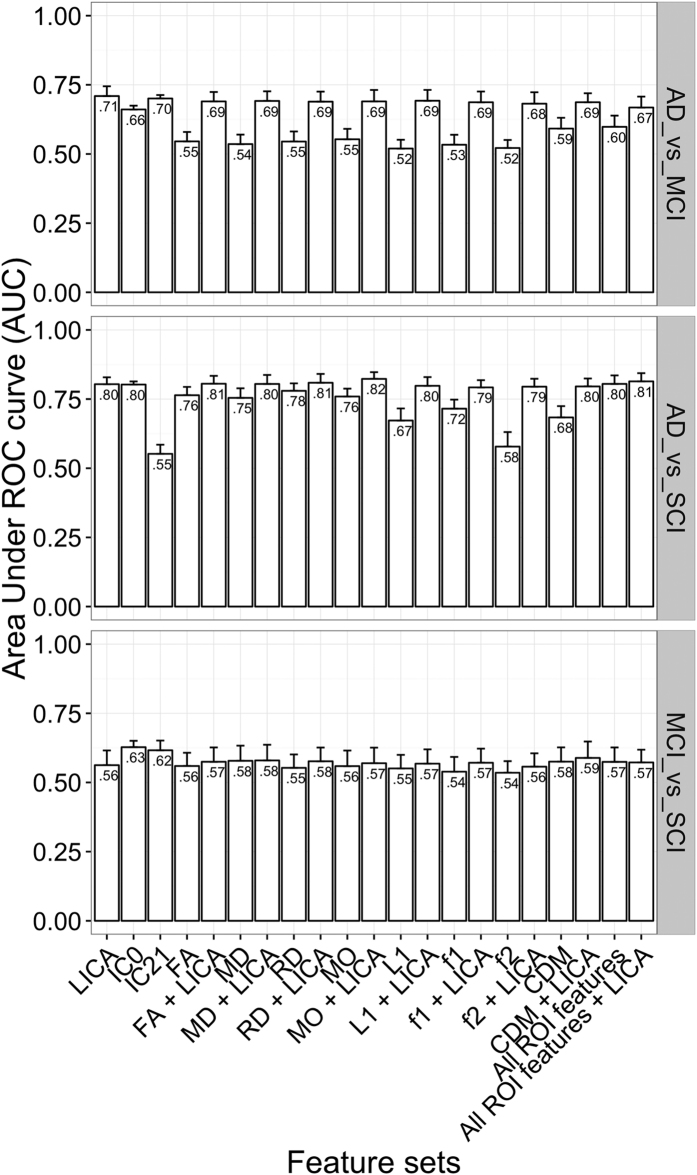
Classification performance. AUC = Area Under ROC curve. The bars represent the mean AUC across 100 iterations and the upper error bars represent the standard deviation. LICA = feature set comprising all LICA components. All ROI features = the combined set of all ROI features (24 features * 8 diffusion MRI metrics). PCA was applied to the ROI feature sets prior to training a classifier.

Figure 2 shows the results from the group classifications. The detailed results in terms of accuracy, sensitivity and specificity are presented in Supplementary Fig. S8. Briefly, we obtained high classification performance of AD vs. SCI using either all LICA features together or IC0 alone (AUC = 0.80 ± 0.02, accuracy = 0.73, sensitivity = 0.72, specificity = 0.77; AUC = 0.8 ± 0.01, accuracy = 0.70, sensitivity = 0.69, specificity = 0.72, respectively). Using either each of the individual ROI feature set or each set in combination with the LICA features yielded comparable results, except for the case of f2, L1, CDM, f1 and MD which yielded lower performance (AUC = 0.58, 0.67, 0.68, 0.72 and 0.75 respectively). While comparable results were obtained for AD vs. SCI regardless of the feature sets used, for the classification of AD vs. MCI, the LICA features provided the highest performance (AUC = 0.71 ± 0.04, accuracy = 0.66, sensitivity = 0.65, specificity = 0.67), which was considerably higher (difference in AUC values ranging from 12% to 19%) than the performance obtained using each of the ROI feature set or a combination of all of them (0.52 < AUC < 0.60). Using IC21 alone yielded higher performance for AD vs. MCI (AUC = 0.7) and MCI vs. SCI (AUC = 0.62), but lower performance for AD vs. SCI (AUC = 0.55) compared to using the ROI features. Furthermore, adding LICA features to the ROI feature set boosted the performance with an improvement ranging from 10% to 17% in AUC values. For the classification of MCI vs. SCI, except IC0 that showed a modest performance (AUC = 0.63, accuracy = 0.57, sensitivity = 0.55, specificity = 0.62), all other feature sets or their combinations resulted in lower performance (0.54 < AUC < 0.58).

When comparing the classification probability obtained using all LICA features, for the classification of AD vs. MCI, the results show a trend that MCI non-carriers of APOE ε4 (n = 23) were given a higher probability of being correctly classified as MCI than the carriers (n = 18, Cohen’s *d* = 0.55, *t* = 1.62, *p* = 0.12, Supplementary Fig. S9). A higher proportion of MCI carriers was misclassified as AD compared to the non-carriers (42% vs. 26%). Similarly, within AD, APOE ε4 carriers (n = 41) were assigned higher probability of being classified as AD than the non-carriers (n = 13, Cohen’s *d* = 0.40, *t* = 1.37, *p* = 0.18, Supplementary Fig. S9). The AD carriers showed a lower proportion of misclassification than the non-carriers (31% vs. 40%). For classification of AD vs. SCI, there was no difference between the non-carrier and carrier groups within either AD or SCI.

When adding APOE ε4 status as an additional feature to the imaging LICA features, the performance was significantly improved across all classifications of AD vs. MCI (AUC = 0.677, LICA only 0.669, *p* = 0.045) and AD vs. SCI (AUC = 0.883, LICA only 0.854, *p* = 0.0003), but not MCI vs. SCI (AUC = 0.595, LICA only 0.590, *p* = 0.19), Supplementary Fig. 10).

## Discussion

Whereas the canonical brain signature of AD has historically and primarily been characterized by medial temporal lobe gray matter atrophy, accumulating evidence has implicated WM microstructural and connectivity abnormalities among the imaging biomarkers showing promise for early risk detection. Here, by fusing eight complementary diffusion MRI metrics of WM anisotropy, diffusivity and connectivity, we identified distinct multimodal WM patterns showing high sensitivity to AD. The primary component, which explained almost 50% of variance in the data captured covariance patterns of all metrics included. The anatomical distribution of this mode of variation is overlapping with previous DTI studies employing univariate approaches^25^,^31^,^32^ and with our results obtained from univariate analysis of the same sample. Importantly, the primary LICA component (IC0) captured a more spatially widespread and bidirectional pattern and yielded greater effect sizes and higher group classification accuracy than conventional ROI based analyses from the same data set, suggesting that LICA is a sensitive approach to the study of WM differences in patient cohorts including AD. IC0 captured the common variance across all eight diffusion MRI measures, reflecting a combination of global and regional WM microstructural and connectivity characteristics. Diffusivity measures (L1, RD, MD), which contributed in total 33% of the overall component weight, reflected a global pattern of increasing diffusivity with advancing cognitive impairment (AD > MCI > SCI), which corresponds with previous meta-analyses of DTI studies in AD and MCI^18^,^19^,^31^.

In contrast to the unidirectional diffusivity pattern discussed above, the measures of anisotropy (FA, MO) showed bidirectional patterns. First, we observed a familiar pattern of large-scale decreased anisotropy, in line with results from conventional voxel-wise analyses of DTI maps from the same dataset (Supplementary Fig. S6), and previous reports^18^,^31^. Second, IC0 also included regions of increased anisotropy (FA, MO) in AD. The pattern of increased FA and MO was largely seen in areas encompassing projection fibers, which is remarkably consistent with previous findings of increased anisotropy in regions of crossing association and projecting fibers in AD and MCI^26^,^33^. Visual inspection of the distribution of the different diffusion MRI measures in the present component corroborated this finding by showing relative sparing of the dominant fiber direction (f1) in a region indicating increased FA in AD and MCI, encompassing both probabilistic atlas renderings of the corticospinal tract and the superior longitudinal fasciculus.

IC0 also involved CDM (5% weight contribution, Fig. 1a), which provided a unique feature of WM connectivity based on full brain voxel-wise probabilistic tractography based on a crossing-fiber model. The CDM pattern was also bidirectional showing both increased and decreased connectivity probability in AD. Specifically, we observed reduced connection density in AD in association pathways with known involvement in the disease, including the fornices^34^ and the uncinate fasciculi^35^. The same pattern was also seen in the genu of the corpus callosum. Conversely, a pattern of increased connection density in AD (AD > MCI > SCI) was restricted to projection pathways, following motor-related pathways including corticospinal tracts bilaterally. We interpret the focal pattern of increased CDM in AD seen here as further support of relative sparing of projecting fibers in early AD and MCI^25^, and may lend support to the retrogenesis model of AD^7^. Although there is much debate on the pathophysiology underlying AD-related WM changes, the retrogenesis hypothesis states that oligodendrocytes and myelin homeostasis are viewed as upstream players in aging and AD-related cognitive decline^7^. According to the model, later-myelinating neurons such as those of limbic association pathways^34^,^35^, are more susceptible to environmental and metabolic insults compared with tracts that myelinate early in ontogeny, such as the corticospinal pathways. In line with this model, Stricker *et al*. found reduced FA in late-myelinating regions within the corpus callosum and in the body of the corpus callosum in MCI compared to healthy elderly controls^36^. Our finding of greater FA reductions and RD increases in AD in the genu of the corpus callosum as compared to the splenium (may be seen in Fig. 1, axial views), also fits with retrogenesis theory, although our study was not designed to evaluate neurobiological properties underlying the diffusion MRI metrics.

The many shared features of AD and normal aging remains a challenge in brain imaging studies of AD^37^. Identification of patterns unrelated to chronological age would be beneficial for better classifying patients with the disease. Interestingly, LICA identified a disease-related independent component (IC21) encompassing anterior callosal and internal capsule connection density (Supplementary Fig. S5), which was essentially age-independent. IC21 classified well between AD and MCI patients, and interestingly showed no relationship with SCI. We speculate that the pattern of IC21 could be interpreted as AD patients depending more heavily on preserved white matter tracts, such as bihemispheric frontal connections and projecting fibers as opposed to more affected, e.g., limbic systems. Increased internal capsule connectivity in AD fits well with the consistent sparing of this tract in AD found in DTI meta-analysis^18^.

Considering all LICA features combined, we observed fairly good performance for the classification between AD and SCI (AUC = 0.80). This performance is comparable if not higher than that obtained using ROI features obtained from the individual measures. Furthermore, LICA features outperformed all ROI feature sets in terms of classification between AD and MCI, and adding LICA features to the ROI feature sets boosted the classification performance (from 10% to 17% increase in AUC value). This indicates that data-driven fusion of multiple diffusion MRI metrics using LICA produces features that are not only biologically interpretable, but also shows increased sensitivity to the differences between AD and MCI. It is also worth noting that such an increased performance in discriminating AD vs. MCI was obtained while maintaining a comparable and high sensitivity for AD vs. SCI with regard to the univariate ROI features. Classification between MCI and SCI was at chance level using either all LICA features or each of the ROI feature sets, although still with the highest yet modest performance obtained by using IC0 as the only feature (AUC = 0.63). The low classification accuracy for MCI vs. SCI is in line with the considerable overlap of demographic and cognitive variables for these two groups.

MCI APOE ε4 carriers were more likely to be classified as AD than MCI non-carriers, and vice versa, AD non-carriers were more likely to be classified as MCI than AD carriers, although only at a trend level (Supplementary Fig. S9). This finding indicates that MCI carriers of the APOE ε4 allele, who are likely at increased risk of progressing to AD compared to their ε4 non-carrier counterparts, also show increased AD-related WM alterations compared to the non-carriers. Further, permutation testing showed that adding APOE ε4 status significantly improved classification performance of AD vs. MCI and SCI. These results may support earlier reports of altered WM microstructure, e.g., reduced FA^14^,^15^ and increased MD as well as RD^13^,^14^, in ε4 carriers relative to non-carriers, and supports that APOE ε4 is associated with characteristic AD-like biomarker profiles also in healthy and sub-clinical populations^12^. However, since the carriers on average were older than the non-carriers within MCI, we emphasize that these results should be interpreted with caution since we cannot fully rule out the confounding effect of age on the results, and further verification in larger, age-matched samples is needed.

Although no previous diffusion MRI study has assessed classification performance on a clinical sample including SCI patients, comparable performance for FA and MD (AUC = 0.79 and 0.83, respectively) has been reported when classifying AD patients and healthy controls using a unimodal approach^22^. In another study which classified 14 amyloid-positive AD patients and 14 closely matched healthy controls, the authors obtained an AUC of 0.87 for five DTI measures^38^, which is higher than the present performance.

When comparing the present results to previous literature, it should be noted that instead of a healthy control group we recruited SCI patients from a memory clinic as a reference group for comparison with AD and MCI. SCI might be a harbinger of early AD pathology^39^,^40^ and our SCI sample likely differ from normal controls without subjective memory problems, potentially contributing to lower classification accuracy than one would obtain from studying a healthy convenience sample.

Among the strengths of our study is the inclusion of naturalistic sample of memory clinic patients with SCI, MCI, and AD, which enabled us to assess common and unique features across a clinically defined dementia/AD spectrum in a relevant setting. To the best of our knowledge, this is the largest diffusion MRI study comparing SCI, MCI and AD dementia patients. Further, the use of LICA allowed us to simultaneously model global and local independent effects across different complementary WM microstructural and connectivity measures, including sensitive indices of crossing fibers and tractography based structural connectivity, which provided a novel view of WM structure in dementia. Since a main advantage of LICA is the ability to model shared variance across different measures, the derived components may show increased sensitivity to an effect of interest, especially in the case when the effect is subtle and present across different measures^41^. This is strongly demonstrated by the increased sensitivity in differentiating AD and MCI offered by the LICA features in the present study.

There are limitations that should be taken into account. According to cortical disconnection theory of aging, WM deterioration including myelin-breakdown, has a mediating role in cognitive decline associated with advancing age^42^. When evaluating disease-related differences associated with cognitive decline in the elderly—for instance between MCI and AD—comparable age groups may reduce confounding. Here, the AD group was not perfectly age-matched with the other groups, making it difficult to completely rule out effects of age in the group comparisons, although age was covaried in the univariate analyses and accounted for by using the residualized features in classification. Since age is the single most important risk factor for AD, the slightly higher age in the AD group is not surprising. Follow-up studies are needed to assess the value for predicting clinical conversion in MCI and SCI patients, and to better disentangle the spurious relationship between healthy and pathological aging. Also, due to the naturalistic setting of recruitment, age-matched controls without memory complaints were not available for the present study. Future research should address the usefulness of LICA on diffusion MRI measures in discriminating the earliest stages of cognitive impairment (e.g., SCI and MCI) from normal controls in the same age range, preferably in a longitudinal setting.

Two different head coils were used during acquisition, which may lead to unwanted source of MRI signal variation. However, the effects of head coil on the WM measures were largely accounted for by one component (IC3), leaving the remaining components largely unaffected (Supplementary Fig. S1). This result provided empirical evidence on the ability of LICA to model and isolate the effect of MR hardware discrepancy into a limited set of independent components.

Another limitation is that we included diffusion MRI data only with a focus on multivariate characterization of WM microstructure and connectivity. LICA however is able to fuse data across MR imaging modalities, and more research is needed in order to assess whether including other indices of AD-related pathology such as amyloid positron emission tomography, cortical and subcortical morphometry could reveal novel multimodal AD patterns and improve classification. Furthermore, we used diffusion measures derived using standard single-shell diffusion weighted imaging. Whereas these measures have been suggested as sensitive features of WM microstructure, changes in their values may not be attributed to changes in specific tissue microstructure substrates^23^. NODDI and RSI are complementary approaches based on multi-compartment models of diffusion^23^,^43^, and may provide microstructural indices with higher specificity compared to conventional diffusion MRI metrics^23^,^44^. However, the application of such multi-compartment models on the single-shell data included in this study, although technically feasible^45^, is suboptimal^23^, and future work should strongly consider additionally including complementary diffusion measures derived using multiple-shell diffusion MRI data.

Lastly, whereas the diagnostics workup was performed by experienced physicians according to research criteria and a comprehensive standardized protocol^46^, the clinical and biological phenotyping (e.g., amyloid and tau status) was limited. Further studies are needed to test for associations with a more complete range of biological, clinical and cognitive phenotypes.

By means of advanced diffusion MRI analysis, we report a multivariate pattern encompassing a wide range of WM microstructural and connectivity information. The data-driven approach revealed a spatial pattern which was highly sensitive to diagnostic status—outperforming a conventional approach to imaging analysis in terms of effect sizes and group classification performance. The results obtained from our novel combination of complementary diffusion MRI metrics strengthen the view that the pathophysiological processes in the AD spectrum are associated with WM structural aberrations with a magnitude closely reflecting clinical severity. Further, we have demonstrated that LICA identifies biological and clinical relevant brain features in a neurodegenerative context, and we argue that future studies should aim to employ multivariate analysis such as LICA as a way to improve AD biomarker development.

## Materials and Methods

### Participant recruitment and screening

Table 1 summarizes demographic and clinical information. We evaluated 217 patients whom had undergone MRI between 2010 and 2014 as part of enrollment in the “Norwegian registry for persons being evaluated for cognitive symptoms in specialized health care (NorCog)”. NorCog is a national patient registry comprising consecutively enrolled patients referred to one of 27 participating memory outpatient clinics for assessment of suspected cognitive impairment or dementia. Included patients were recruited from the memory clinic at Oslo University Hospital, which is the largest NorCog center. Eligible patients were assessed in accordance with a standardized clinical examination protocol^46^. Two experienced memory clinic physicians diagnosed the patients according to research criteria in consensus (K.E./A.B., or M.L.B./K.P.). Inclusion criteria included a diagnosis of either AD dementia based on ICD-10 criteria for research N = 137^47^, MCI based on the Winblad criteria N = 78^48^, or patients referred with a subjective cognitive complaint that did not fulfill MCI or dementia criteria, termed SCI N = 38^49^. Thirty eight patients with a diagnosis other than AD, SCI and MCI were excluded, including mixed dementia (n = 10), vascular dementia (n = 5), probable Parkinson’s disease with dementia or Lewy-body dementia (n = 8), fronto-temporal dementia variants (n = 5), or a primary psychiatric disorder (n = 10). Degree of global cognitive impairment was quantified using Mini Mental State Examination (MMSE)^50^. More extensive neuropsychological evaluation, including tests of episodic memory, processing speed and executive function was available for a subset of patients as reported in Supplementary Table S1.

APOE status was available from a subset of the patients (AD = 54, MCI = 41, SCI = 23, see Table 1 for allelic distribution within groups). APOE typing was done using the Illunina Infinium OmniExpress v1.1 chip at deCODE Genetics, Reykjavik, Iceland. We combined subjects carrying at least one copy of APOE ε4 allele, which confers increased risk of AD, in one group, referred to as the APOE ε4 carrier group (n = 63), and all the other subjects in another group, referred to as the non-carrier group (n = 55). The proportion of APOE ε4 carriers in AD was higher than in MCI and SCI. There were no age differences between the carrier and non-carrier groups within AD or SCI. Within MCI, the carrier group was older than the non-carriers.

### Research ethics

The Regional Committee for Medical Research Ethics in South-Eastern Norway approved the study (Reference number: 2013/2283). The study was performed strictly in accordance with the approved guidelines and regulations. All participants gave written informed consent. Patients were only enrolled if determined to have capacity for consent by the evaluating physician.

### MRI acquisition and quality control

A 3 Tesla GE Signa HDxT scanner at Oslo University Hospital was used to collect MR data using two different head coils (standard 8-channel head coil (8HRBRAIN) or Head/Neck/Spine (HNS) coil, *N* per group is given in Table 1). For diffusion weighted imaging, a 2D spin-echo whole-brain echo planar imaging pulse with the following parameters was used: Repetition time (TR) = 15 s; echo time (TE) = 85 ms; flip angle = 90°; slice thickness = 2.5 mm; field of view (FOV) = 240 × 240; acquisition matrix = 128 × 128; in-plane resolution = 1.875 * 1.875; 30 volumes with different gradient directions (b = 1000 s/mm^2^) and two b = 0 volumes with reversed phase-encode (blip up/down) were acquired, yielding pairs of images with distortions going in opposite directions. Image analyses were done using FSL^51^,^52^,^53^, including *topup* (http://fsl.fmrib.ox.ac.uk/fsl/fslwiki/TOPUP) and *eddy* (http://fsl.fmrib.ox.ac.uk/fsl/fslwiki/EDDY)^54^ to correct for subject motion, geometrical distortions by using blip up/down volumes, and eddy currents. The *B*-matrix was reoriented accordingly^55^. *Eddy* was also used for automated identification of slices with signal loss and replace them by non-parametric predictions using Gaussian Process^56^, resulting in a significant improvement in temporal signal-to-noise ratio^57^ (tSNR, Supplementary Information (SI), Supplementary Fig. S11).

Quality control (QC) was performed using both automated and manual procedures. We excluded 10 datasets (5 AD, 3 MCI, 2 SCI) due to insufficient brain coverage (portions of either superior or inferior cerebrum and/or cerebellum were excluded in the acquisition). Five additional datasets were flagged due to low tSNR^57^ and discarded after careful visual evaluation. 164 datasets (30 SCI, 55 MCI, and 79 AD) passed QC and were included in the analysis detailed below.

### Image processing

Eight complementary diffusion MRI indices of the WM microstructure and connectivity were generated for each participant (Supplementary Fig. S12 shows a sample view from one representative dataset). In particular, using a tensor-model fit in FSL, we derived two measures of voxel diffusion anisotropy (fractional anisotropy (FA) and the mode (MO) of the diffusion tensor^58^) and three diffusivity indices (mean (MD, average of the three eigenvalues), axial (L1, the primary eigenvalue) and radial (RD, mean of the second and third eigenvalues) diffusivity).

Two additional crossing-fiber measures were calculated by fitting a two-fiber diffusion model (bedpostX), referred to as the partial volume or ball-and-stick model^59^,^60^,^61^. The multi-fiber model used here yielded an estimated volume fraction of the dominant (f1) and the non-dominant (f2) fiber orientation, reflecting the diffusion strength along the dominant and non-dominant axes of the diffusion tensor, respectively.

Finally, we calculated whole brain connectivity density maps for each participant by means of probabilistic tractography. Specifically, we performed probabilistic fiber tracking using probtrackx2^59^, which samples the voxelwise posterior probability distributions generated by bedpostx. For each participant and from each voxel inside the entire native space whole brain seed mask, 200 streamlines were followed, resulting in a single 3D-volume tractography map per participant, which we then normalized by dividing it with the total number of streamlines processed for each participant. The normalized value in each voxel represents the likelihood that any streamline will pass through that voxel, which was used as a measure of WM connection density (CDM).

To enable voxelwise between-subjects analyses we used tract-based spatial statistics (TBSS)^62^. FA volumes were aligned to the FMRIB58_FA template using nonlinear registration (FNIRT)^63^,^64^. Next, mean FA were derived and thinned to create a mean FA skeleton, representing the center of tracts common across subjects. The same procedures were applied for all other metrics. We thresholded and binarized the mean FA skeleton at FA > 0.2 before the resulting data was fed into voxelwise statistics.

To summarize data in a neuroanatomical framework we calculated mean DTI values across the skeleton and within regions of interest (ROIs) based on the intersection between the skeleton and the probabilistic JHU white-matter atlases^65^,^66^. For each measure, a set of 24 features was derived, hereafter referred to as unimodal ROI feature set as it involves only one DTI map, including 20 ROIs, 4 additional features comprising the mean value across the skeleton, as well as the genu, splenium and body of the corpus callosum as defined by the JHU ICBM-DTI-81 WM labels atlas^67^. ROI data were also used in multivariate classification analyses (see below).

### Fusion of eight diffusion MRI measures using linked independent component analysis

We performed data-driven decomposition of the DTI data using FMRIB’s LICA (FLICA, http://fsl.fmrib.ox.ac.uk/fsl/fslwiki/FLICA), which models the inter-subject variability across measures^28^,^29^. We resampled each of the TBSS skeleton maps to an isotropic 2 mm resolution, in line with^28^, using a nearest neighbor interpolator. The resulting maps derived from all 164 individual DTI datasets, including FA, L1, RD, MD, MO, f1, f2, and CDM were included in the LICA decomposition to model both the common and unique inter-subject variability across the eight metrics.

A model order of 25 was heuristically chosen based on the spatial maps, resulting in a biologically meaningful yet manageable set of patterns. After careful inspection of all components’ spatial maps and subject loadings for potential outliers, one component (IC3) reflecting head coil variability was excluded from further group pairwise comparison and classification analyses. We evaluated the robustness of the LICA components sensitive to diagnosis across different model orders and presented the results in SI. Correlations among the mean skeleton values of eight measures and two components showing strong diagnosis effect can be seen in Supplementary Fig. S13.

### Statistical analyses

#### Assessment of diagnostic group differences in LICA components and conventional voxel-wise diffusion MRI maps

We tested diagnostic group differences in all LICA components using general linear model (GLM) on the components’ subject loadings, accounting for age, sex and head coil. For the components showing significant group effects, we also tested for associations with MMSE across and within groups. Effect sizes of pairwise group comparisons in subject loadings were standardized using the Cohen’s *d* as follows: *cohen’s* 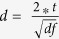, where *t* was the *t* statistics and *df* the degree of freedom of the residuals. We also tested for main effects of age and head coil in the same GLM.

For comparison with LICA results, we also tested for group differences using voxelwise non-parametric analyses of each diffusion MRI map separately by means of GLM in *randomise*^68^. Again, age, sex and head coil were included as covariates. The data was tested against an empirical null distribution generated across 5000 permutations in order to correct for multiple comparisons across space. Threshold free cluster enhancement^69^ was used to avoid manually defining the cluster-forming threshold. Voxel-wise maps were thresholded at *p* < 0.05 (two-tailed), corrected for multiple comparisons across space.

#### Multivariate machine learning classification using LICA and ROI DTI features

To evaluate the discriminative power of the LICA patterns at an individual level, we performed group classification using the *lasso* classifier^70^ in a nested *10*-fold cross-validation framework (see SI for more details). To account for effects of normal aging while avoiding removing disease-related effects associated with advancing age^71^,^72^, using GLM we estimated age effects using SCI subjects only and computed the residuals of each feature on all datasets (scatter plot of the raw and residualized subject loadings of IC0 can be seen in Supplementary Fig. S14). We assessed the classifier performance using area under the receiver-operator characteristics curve (AUC), accuracy, specificity and sensitivity.

Additionally, to compare the discriminative power in pairwise group classification between LICA features and each of the univariate ROI measures (FA, L1, RD, MD, MO, f1, f2 and CDM, 24 features per metric), we applied the same classification framework as described above using each of the conventional ROI feature sets as features. We also evaluated the classification performance using each of the ROI feature set together with the LICA features, and using all features together. Unlike the LICA features, which were only weakly correlated (maximal magnitude of *r* = 0.29), several of the ROI features are highly inter-correlated. To remove this inter-correlation, which could hinder classifier stability and performance^73^, we applied principle component analysis (PCA) on the ROI features prior to classification (more details in SI). Finally, to evaluate the discriminate power in group classification of the LICA component showing the strongest group effect, we ran the same *10*-fold classification framework using a regular linear discriminate classifier^74^ and this component as the only feature.

To assess whether the classification performance differed between APOE ε4 carrier and non-carriers within each group, we compared the prediction probability, bounded between 0 and 1, which quantified the certainty when assigning a class label to a given dataset during testing. In particular, for the classification between AD and MCI, within each group, we compared the ε4 carriers and non-carriers in terms of the probability of being assigned the correct group label, as well as the proportion of the misclassified subjects using GLM, accounting for age, sex and head coil. The same analysis was performed for the classification AD vs. SCI.

Lastly, on the subset for which both imaging data and APOE ε4 information were available, we performed pairwise group classification with APOE ε4 status included as an additional feature and used permutation testing with 10000 iterations to evaluate if the performance was improved when additionally using the genetic feature in combination with the imaging-based LICA features (see SI for details).

LICA decomposition was performed in Matlab (version R2014a). All statistical and machine learning analyses were performed in R (http://cran.r-project.org, version 3.2.1). The *glmnet*^70^ and *caret*^75^ R packages were used for classification and the *ggplot2* package^76^ for visualization. For the pairwise group comparisons of the LICA features, we corrected for multiple comparisons using the Bonferroni procedure (multiplying the p-value by a factor of 72). We used a significance threshold of 0.05.

## Additional Information

**How to cite this article:** Doan, N. T. *et al*. Dissociable diffusion MRI patterns of white matter microstructure and connectivity in Alzheimer’s disease spectrum. *Sci. Rep.* **7**, 45131; doi: 10.1038/srep45131 (2017).

**Publisher's note:** Springer Nature remains neutral with regard to jurisdictional claims in published maps and institutional affiliations.

## Supplementary Material

Supplementary Information

## Figures and Tables

**Table 1:** Sample characteristics.

	SCI (n = 30)	MCI (n = 55)	AD (N = 79)	Group differences
Age	63.0 ± 9.5	65.5 ± 11.2	70.2 ± 8.1	*(AD > MCI; AD > SCI)
Gender, % females	53	36	58	*(AD_♀_ > MCI_♀_)
MMSE§, score	29.4 ± 0.6	28.0 ± 2.2	22.3 ± 5.4	*(AD < MCI; AD < SCI)
Education§§, years	15.0 ± 3.9	14.0 ± 3.2	12.2 ± 3.2	*(AD < MCI; AD < SCI)
Heal coil, % 8HRBRAIN	67	69	62	No significant group effect
APOE status *N* (% ε4+†)	23 (17)	41 (44)	54 (76)	*(AD_ε4+_ > MCI_ε4+_ > SCI_ε4+_)

^§^MMSE missing for 5 MCI, and 2 SCI.

^§§^Years of education not available for 1 SCI, 4 MCI, 7 AD.

^†^% ε4+ denotes the percentage of participants with known APOE status who had at least one ε4 allele.

^*^Significant main effect of group (*P* < 0.05) based on univariate linear models. “>” and “<” denote significant group differences (*P* < 0.05) in the indicated direction based on post-hoc testing using Bonferroni-correction for three comparisons.
