# Supplementary material for: Dissociable diffusion MRI patterns of white matter microstructure and connectivity in Alzheimer’s disease spectrum
Source: Sci Rep. 2017 Mar 24;7:45131. doi: 10.1038/srep45131 (PMC5364534; doi:10.1038/srep45131)
Supplement: Supplementary Information [file srep45131-s1.pdf]

Dissociable diffusion MRI patterns of white matter microstructure and connectivity in Alzheimer's disease spectrum

Nhat Trung Doan[1*#], Andreas Engvig[1,2#], Karin Persson[3,4], Dag Alnæs[1], Tobias Kaufmann[1], Jaroslav Rokicki[1,5], Aldo Córdova-Palomera[1], Torgeir Moberget[1], Anne Brækhus[3,4], Maria Lage Barca[3,4], Knut Engedal[3,4], Ole A. Andreassen[1], Geir Selbæk[3,6], Lars T. Westlye[1,5]

[1]NORMENT, KG Jebsen Centre for Psychosis Research, Division of Mental Health and Addiction, Oslo University Hospital & Institute of Clinical Medicine, University of Oslo, Norway,

[2]Department of Medicine, Diakonhjemmet hospital, Oslo, Norway,

[3]Norwegian National Advisory Unit on Ageing and Health, Vestfold Hospital Trust, Tønsberg, Norway,

[4]Department of Geriatric Medicine, The Memory Clinic, Oslo University Hospital, Oslo, Norway,

[5]Department of Psychology, University of Oslo, Oslo, Norway

[6]Centre for Old Age Psychiatric Research, Innlandet Hospital Trust, Ottestad, Norway,

# These authors contributed equally

* Correspondence: Email: n.t.doan@medisin.uio.no, Oslo University Hospital, PoBox 4956 Nydalen, 0424 OSLO, Norway, Phone: +47 23 02 73 50

Supplementary Information

Materials and Methods

Effect of eddy slice replacement on temporal signal-to-noise ratio (tSNR)

We used the latest version of FSL's *eddy* tool, which includes a new feature on replacement of slices with signal loss due to subject movement. This resulted in an increase in tSNR by 3%. Results from a repeated ANOVA showed that there were significant main effects of group ($p<0.0001$) and *eddy* version ($p=0.0008$) but there was no significant interaction effect. Supplementary Fig. S11 presents the density plots of tSNR before and after *eddy* slice correction.

Multivariate machine learning classification

We performed group pairwise classification using the *lasso* classifier in a nested *k*-fold classification framework. For each classification, the entire sample was split into *k* partitions of equal size. One partition was left out for validation (a.k.a. the testing set). The classifier was then built using the (*k-1*) remaining partitions, on which another *k*-fold cross-validation was applied to estimate the regularization parameter λ. As the group size was imbalanced in most cases, to alleviate classification bias toward higher accuracy on the majority class, we balanced class size of the training set (the testing set remained untouched), based on a resampling technique as implemented in the *ROSE* R package [1]. The trained classifier was then applied on the left out partition. This process is repeated for each of the partitions. We chose the commonly used value of 10 for *k* [2]. The entire process was repeated 100 times and the average performance (accuracy, specificity, sensitivity, and area under the receiver-operator characteristics curve (AUC)) was computed. All features were standardized via mean centering and scaling to have a standard deviation of 1.

For the ROI DTI feature sets, in each iteration and within each fold, a principle component analysis (PCA) was applied on the training set using the *prcomp* function in R, retaining only the number of components explaining 95% of the data variance for further classification. The learned PCA mapping was then applied to the testing set for evaluation. For comparison purposes, we also reported the classification performance using the raw features (without PCA) as Supplemental Information. A similar performance was observed without the PCA step.

Robustness of multimodal LICA IC0

Supplementary Fig. S4 presented the main effect of group, accounting for age, sex and head coil on IC0 as a function of model order as well as the amount of explained variance (in percentage) that this component captured among all components. Across a range model order (from 15 to 50), IC0 robustly showed strong diagnosis effect ($15.7 < f$ statistics < 16.76). Additionally, despite the expected decreasing amount of explained variance by IC0 as model order increased, this component consistently explained the largest amount of variance among all components for a given model order. These results suggest that IC0 is robust against the choice of model order in the data-driven LICA decomposition.

Testing the added value of APOE ε4 in group classification

We performed classification using the combined APOE ε4 status information and all imaging LICA features and compared with the results obtained using only the LICA features on the same subsample, as follows. We assigned a label of 1 to subjects carrying at least one APOE ε4 and 0 otherwise. This additional feature was then normalised (mean-centering and scaling) the same way as the LICA features prior to classification. We assessed the significance of improvement in performance using permutation testing. In particular, we randomly permuted values of the APOE ε4 feature across all subjects, keeping the LICA features the same, and subsequently run the

classification. This process was repeated 10000 times, resulting in a null distribution of the performance (area under the ROC curve (AUC)). The performance obtained from the non-permuted data was then compared to the null distribution to obtain the *p*-value. We declared significance if the *p*-value was smaller than 0.05. Supplementary Fig. 10 presents the null distribution of AUC as well as the results obtained from the non-permuted data.

References

1 Lunardon, N., Menardi, G. & Torelli, N. ROSE: A Package for Binary Imbalanced Learning. *A peer-reviewed, open-access publication of the R Foundation for Statistical Computing*, 79 (2014).

2 James, G., Witten, D., Hastie, T. & Tibshirani, R. *An introduction to statistical learning: with applications in R*. Vol. 6 (Springer, 2013).

Table S1. Neuropsychological test results from SCI, MCI and AD patients.

	SCI	MCI	AD	Group differences
CERAD 10-word recall, sum of trials[*]	21.6 ± 4.30	17.3 ± 3.98	12.0 ± 4.90	(AD < MCI < SCI)[§]
Trail making test (part A), seconds[**]	34.8 ± 15.2	53.6 ± 29.6	79.7 ± 84.5	(AD > SCI) [§]
Trail making test (part B), seconds[***]	88.6 ± 33.6	118 ±61.5	183 ± 106	(AD > MCI & SCI) [§]

Table footnote: [*]Available for 24 SCI, 37 MCI, and 75 AD. [**]Available for 29 SCI, 50 MCI, and 72 AD. [***]Available for 28 SCI, 41 MCI, and 46 AD. [§] Denotes significant group differences ($P < .05$) in the indicated direction based on post-hoc testing using Bonferroni-correction for three comparisons.

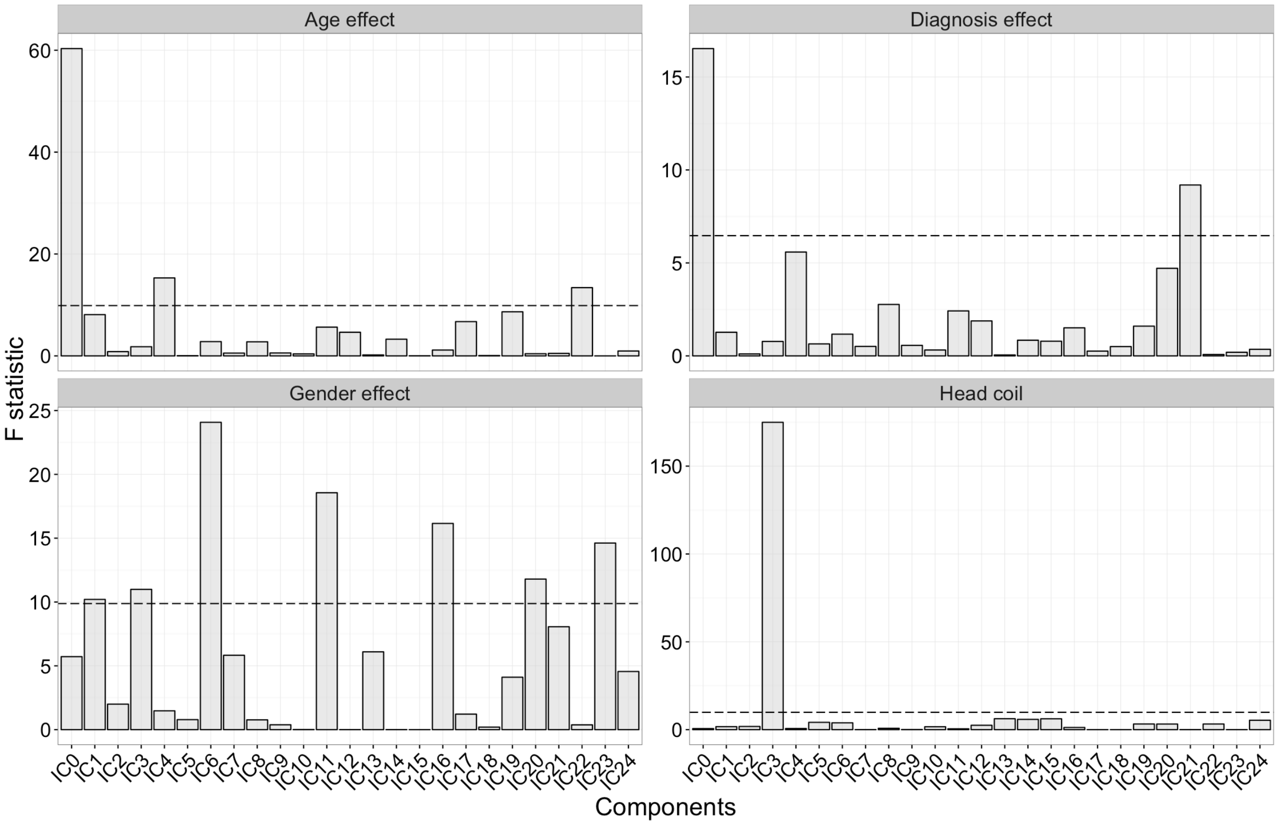

Figure S1. Results of main effect analyses using ANOVA. The dashed line in each subplot represent the significance threshold, corrected for the number of LICA components, obtained using the Bonferroni procedure. The *F*-statistic threshold was computed according to a significance *p*-value of 0.002 (0.05/25) for each effect.

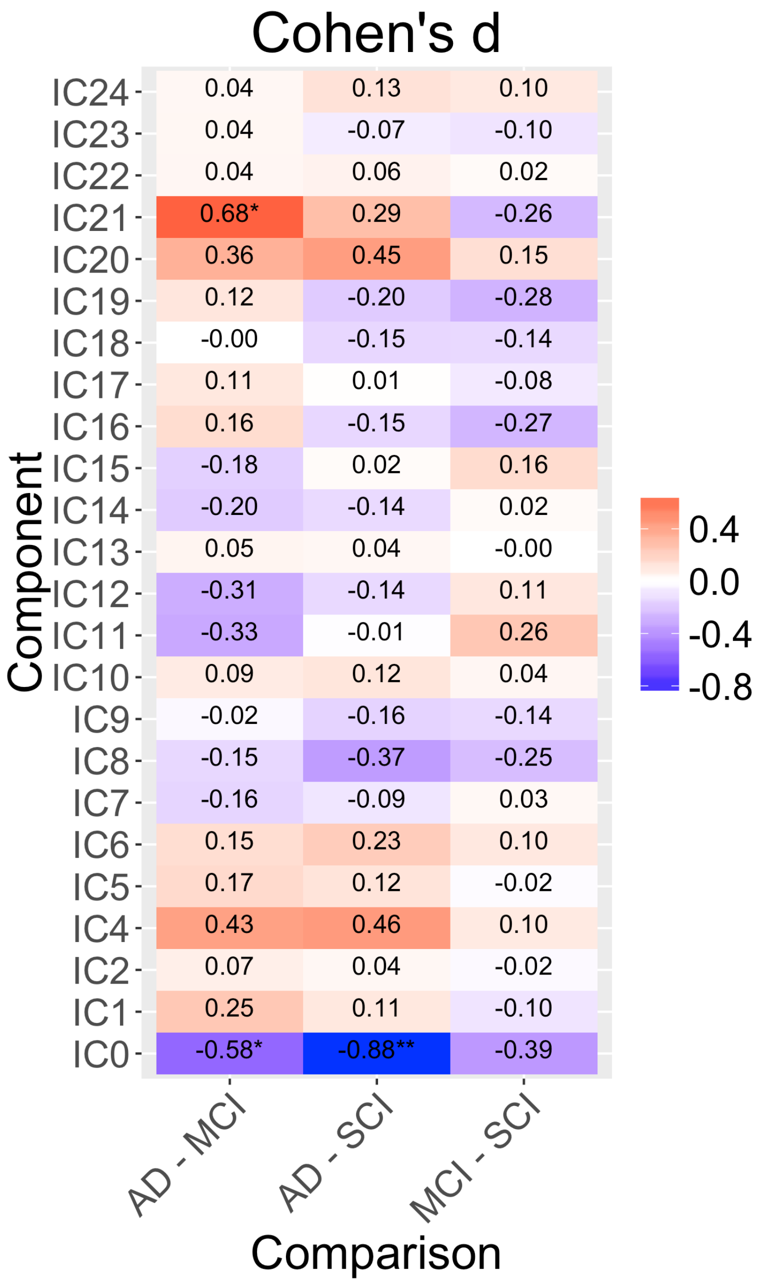

Figure S2. Effect size (Cohen's *d*) of group pairwise comparisons. "*", "**" indicate significance levels (0.001<"*"<0.05, "**"<0.001) after correcting for multiple comparison using the Bonferroni procedure.

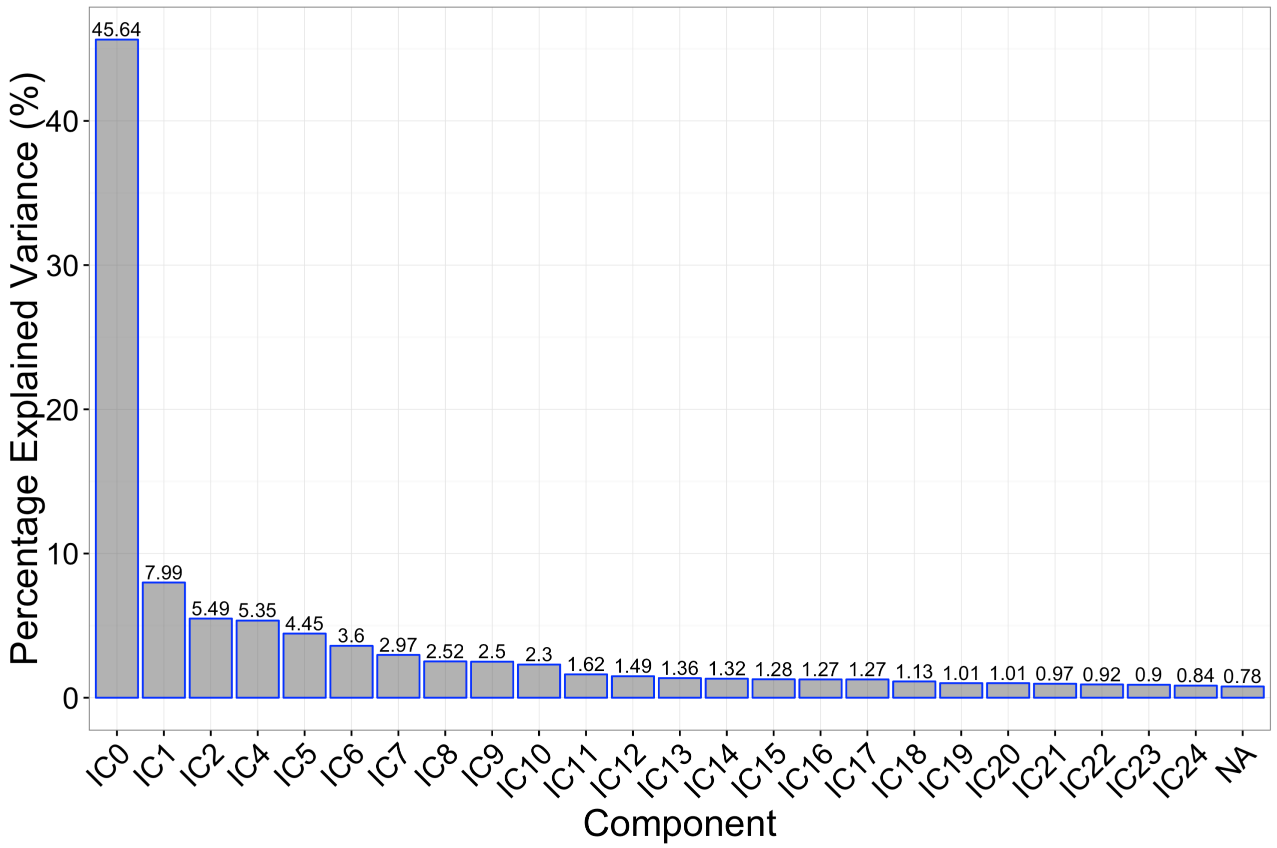

Figure S3. Percentage explained variance of all LICA components.

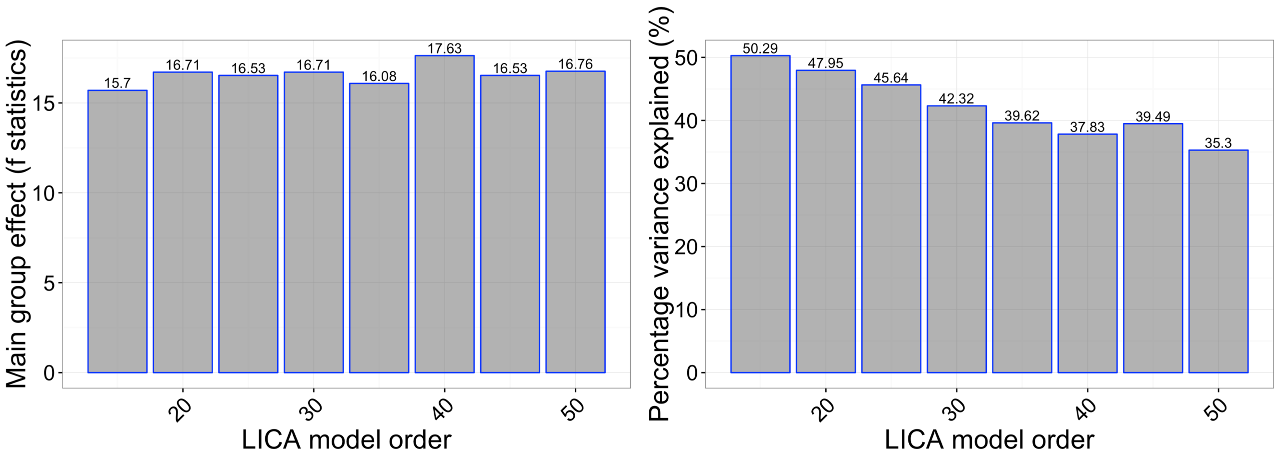

Figure S4. the main effect of group, accounting for age, sex and head coil on IC0 across different model orders as well as the amount of explained variance (in percentage) that this component captured among all components.

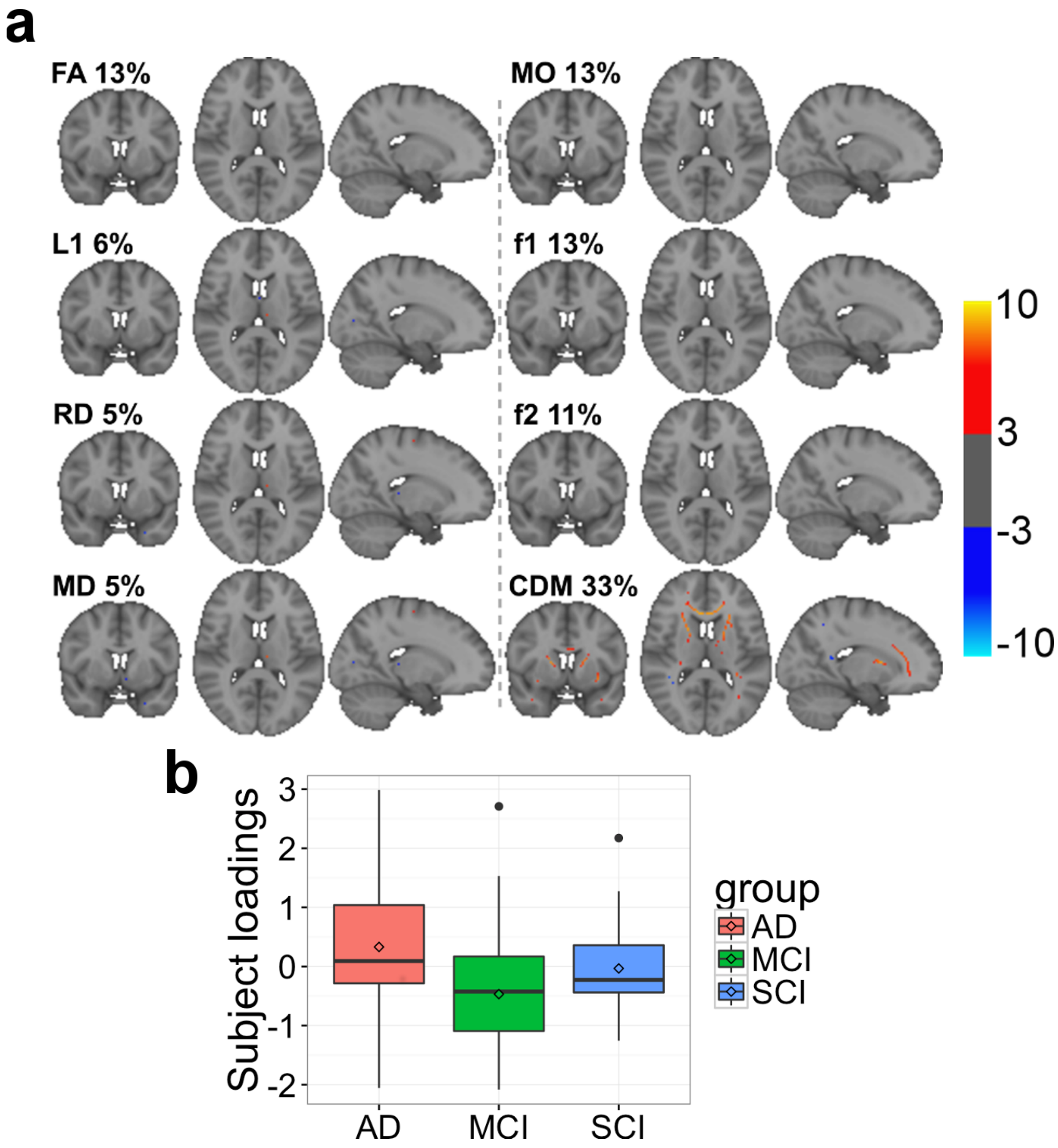

Figure S5. IC21: (a) Spatial maps and (b) subject loading distribution. The spatial maps represent the thresholded z-scores ($3 < |z| < 10$). In the spatial maps, the weights (in percentage) indicate the relative contribution of each measure to the component at the group level. In the subject loading plot, the box represents the 25% and 75% quantiles, the horizontal bar in the box representing the median, the diamond the mean, and the dots representing outliers.

a. AD - SCI

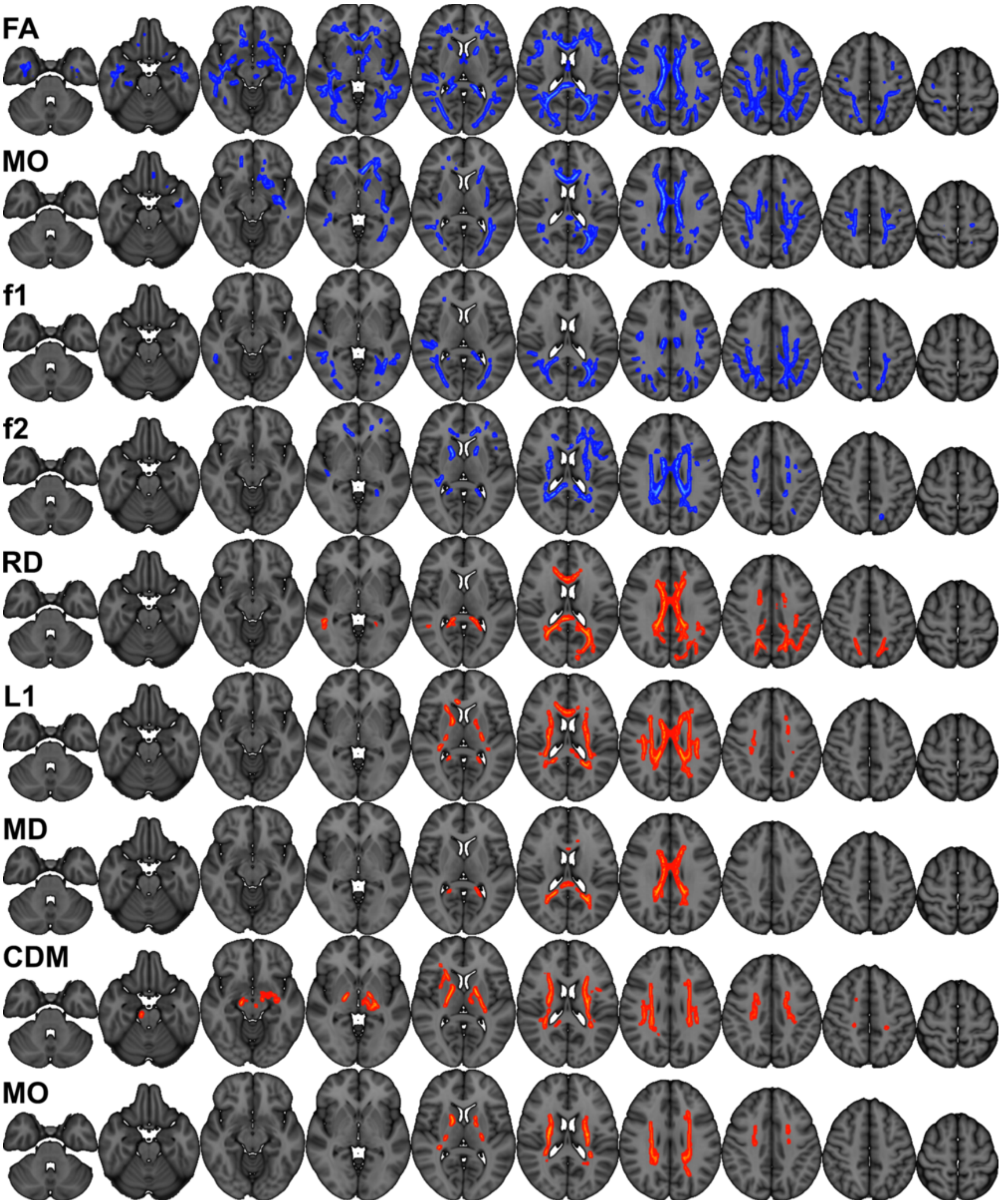

b. AD - MCI

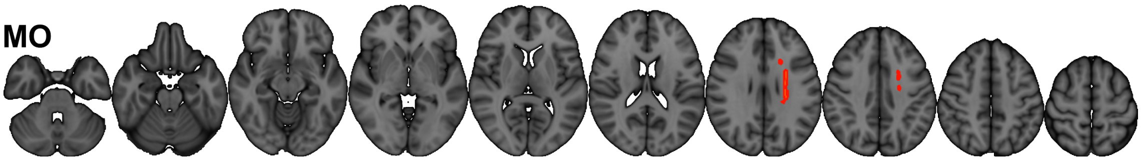

Figure S6. Univariate results: (a) AD vs. SCI, (b) AD vs. MCI. Maps of p-values showing significant difference ($p<0.05$, two-tailed) based on threshold free cluster enhancement (*tfce*). Red and blue colors indicate positive and negative differences, respectively.

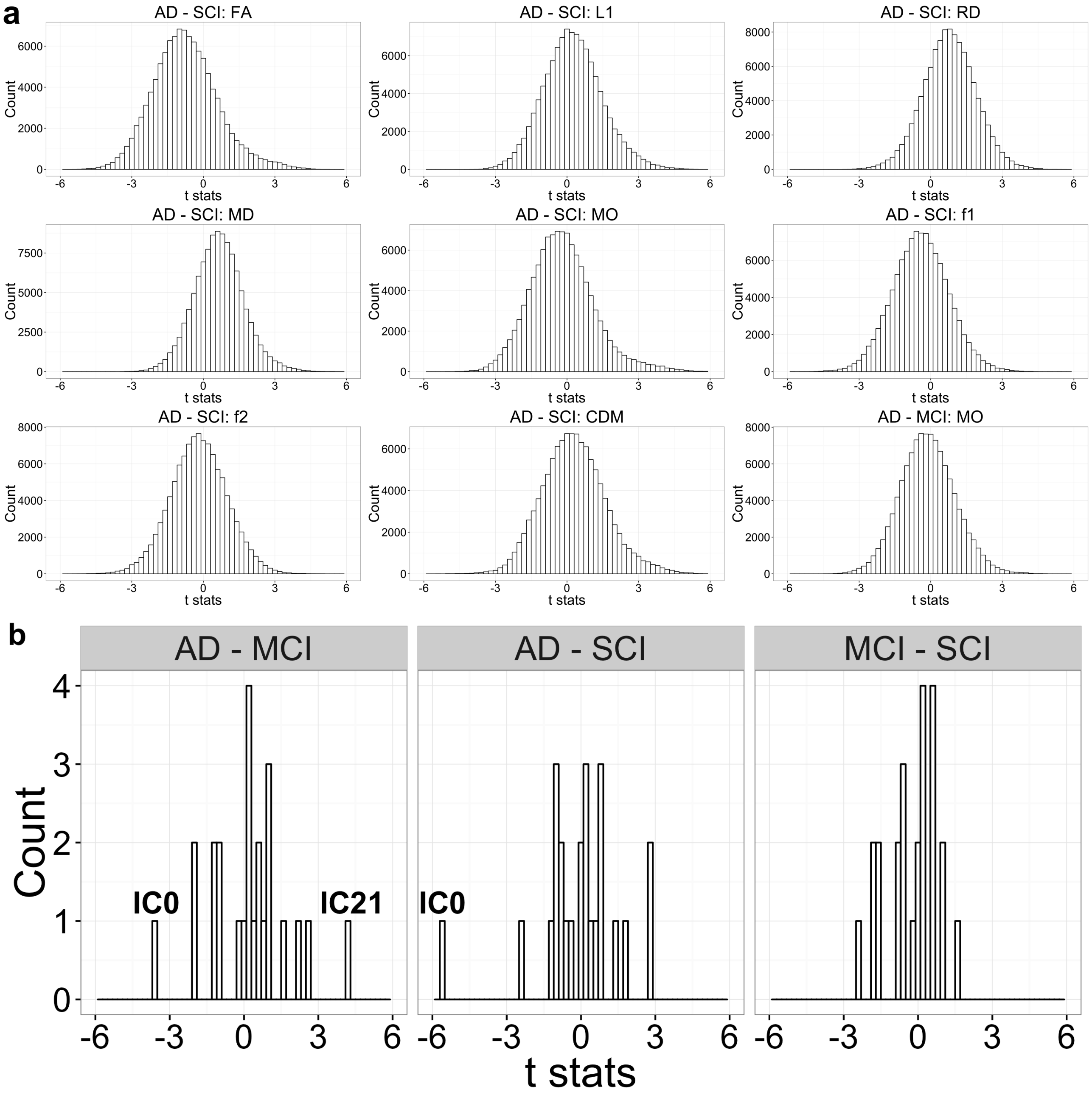

Figure S7. Histogram of the *t*-statistics obtained from univariate analyses of each DTI map (a) and LICA features (b).

a

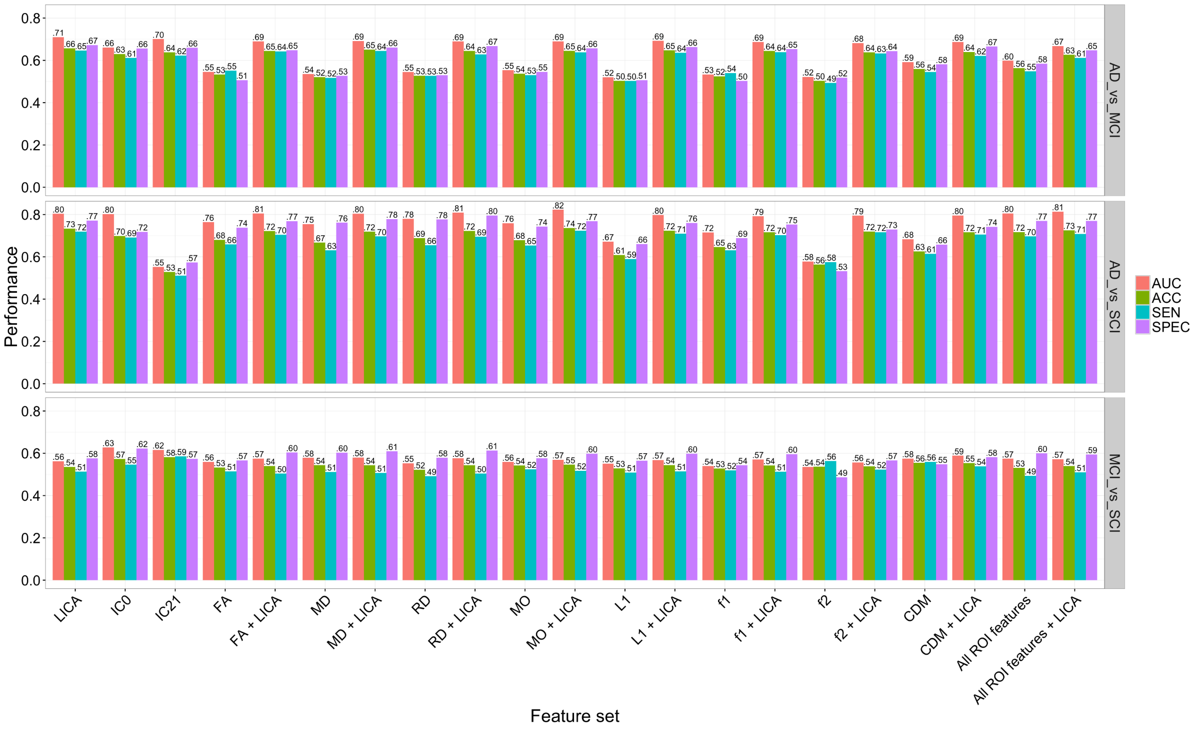

b

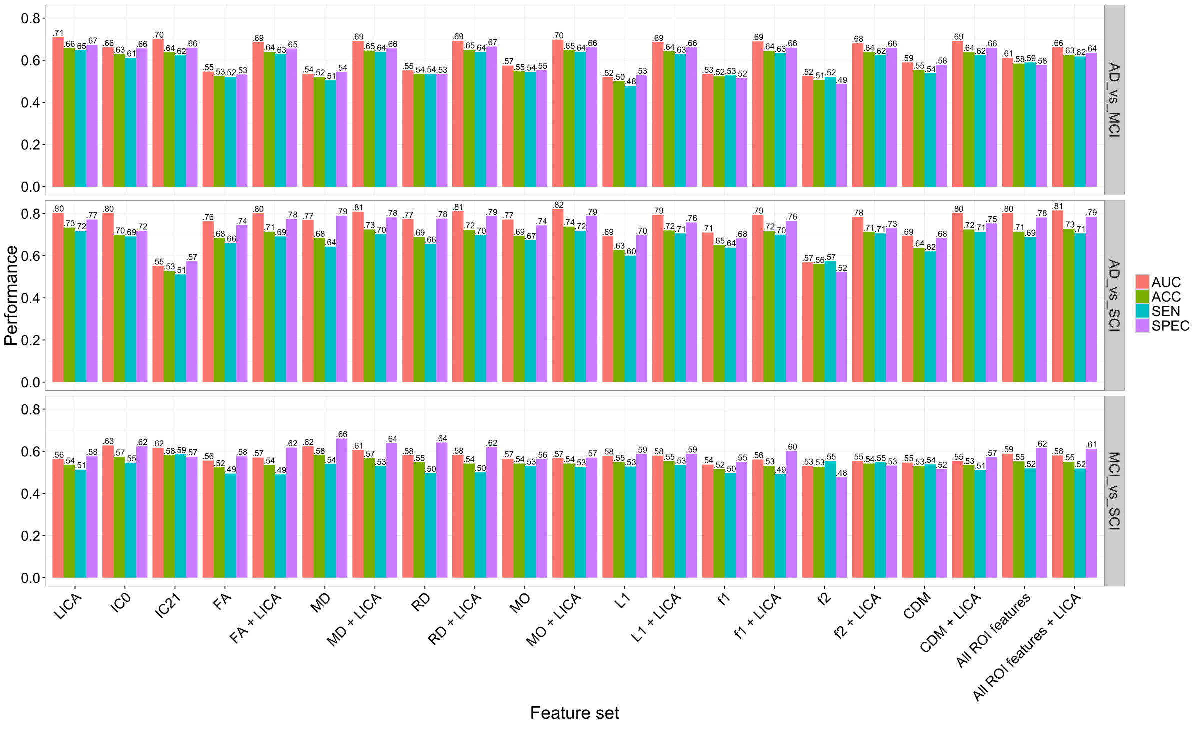

Figure S8. Classification performance: (a) PCA was applied to the unimodal ROI feature sets prior to training a classifier, (b) no PCA was applied during training. AUC = Area Under ROC curve, ACC = Accuracy, SEN = Sensitivity, SPEC = Specificity. LICA = feature set comprising all LICA components. All ROI features = the combined set of all ROI features (24 features * 8 DTI metrics).

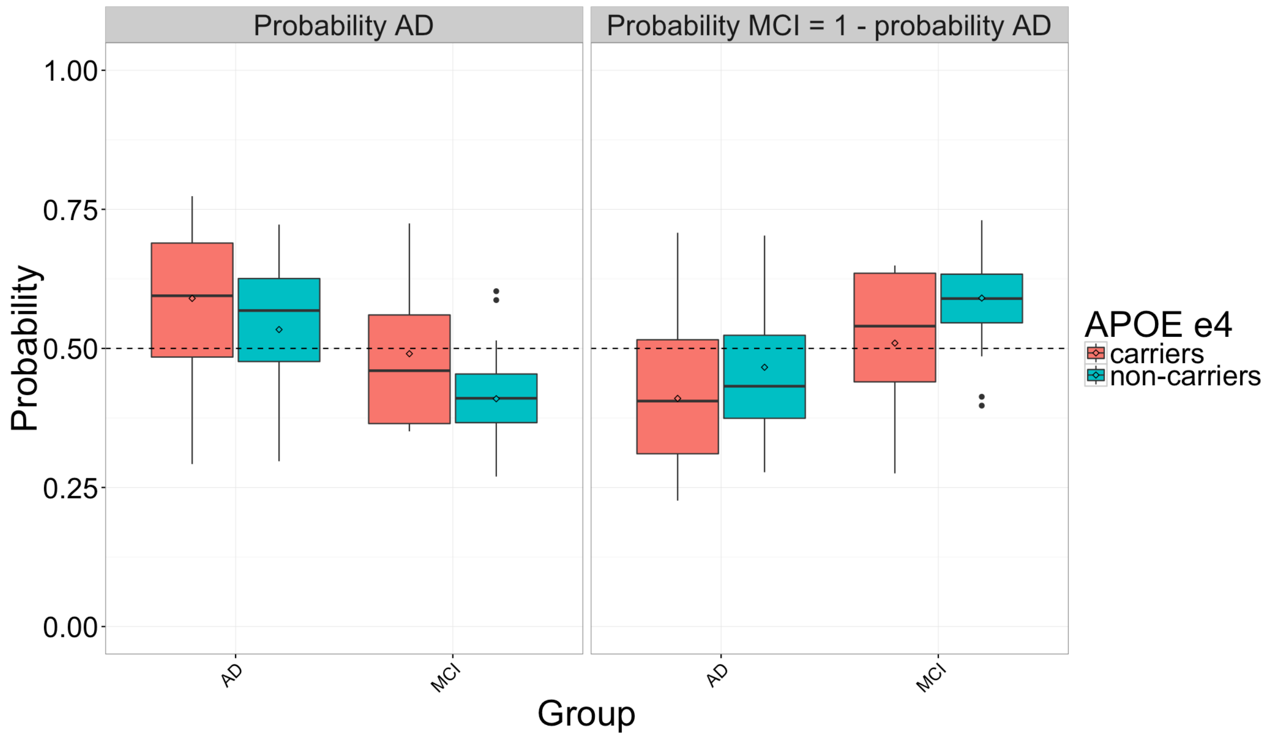

Figure S9. Probability of class labelling for the classification AD vs. MCI. The dashed line represent the chance level. For a given class, the smaller the probability, the less likely the dataset belongs to the corresponding class, and the closer the probability is to 0.5, the less certainty the classifier has in assigning a label. Both the carrier MCI and non-carrier AD groups were closer to the chance level than their counter groups, indicating a higher overlap in terms of WM phenotypes between the carrier MCI and non-carrier AD.

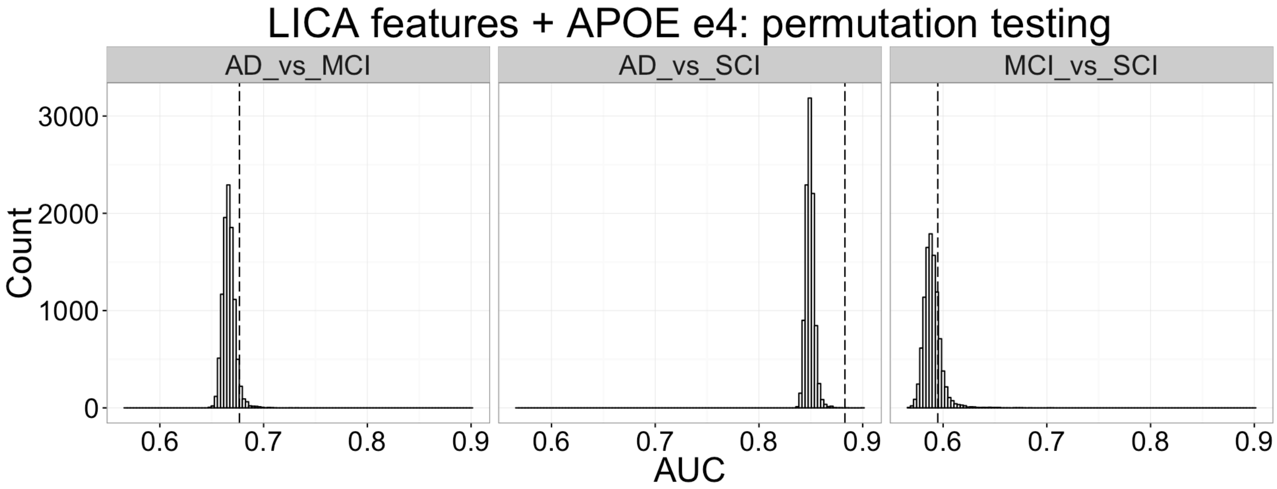

Figure S10. Null distribution of the AUC performance measure obtained by running classification on the combined set of LICA and APOE ε4 features after randomly permuting the APOE ε4 feature with respect to the diagnosis label across all subjects. The LICA features were not permuted. This process was repeated 10000 times for each classification pair. The dashed lines represent the performances (AUC) obtained using the non-permuted LICA and APOE ε4 features.

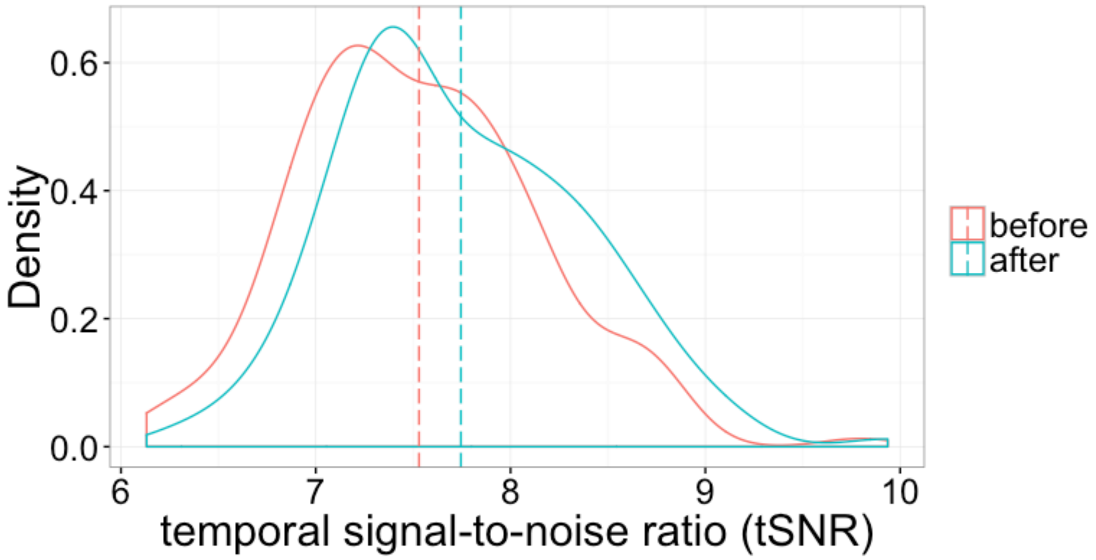

Figure S11. Density plots of temporal signal-to-noise ratio (tSNR) before and after applying *eddy* to identify slices with signal loss due to subject movement and replace them with non-parametric predictions using Gaussian processes. The dashed lines represent the means. A significant improvement in tSNR was gained.

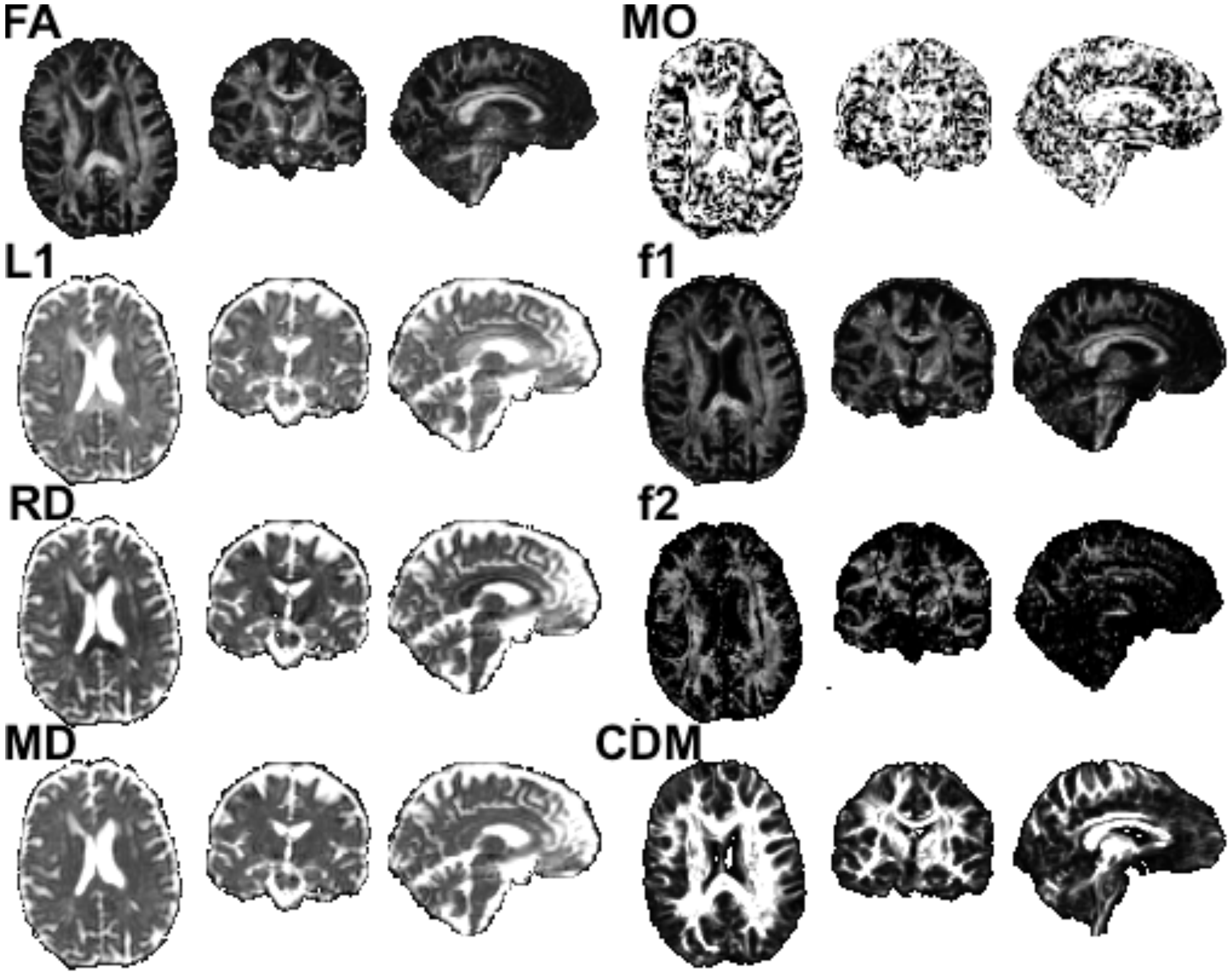

Figure S12. Illustrative example of eight DTI indices derived from a representative dataset. FA = Fractional Anisotropy, L1 = Axial Diffusivity, RD = Radial Diffusivity, MD = Mean Diffusivity, MO = Mode of Diffusion Tensor, f1 = Dominant Fiber Orientation, f2 = Non-dominant Fiber Orientation, CDM = Connectivity Density Map.

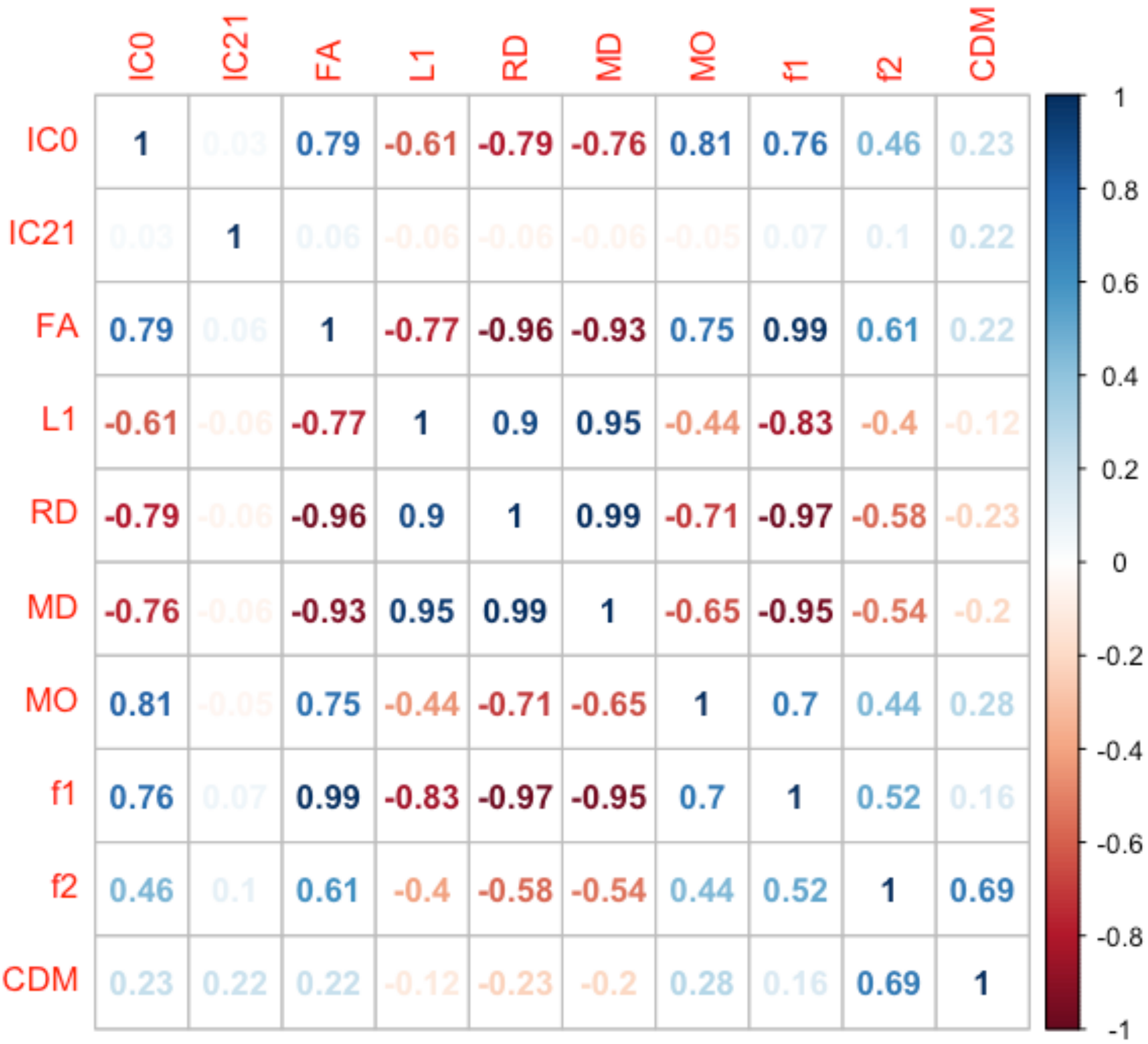

Figure S13. Pairwise correlation between IC0, IC21 and the mean skeleton value of each diffusivity and connectivity-based metric.

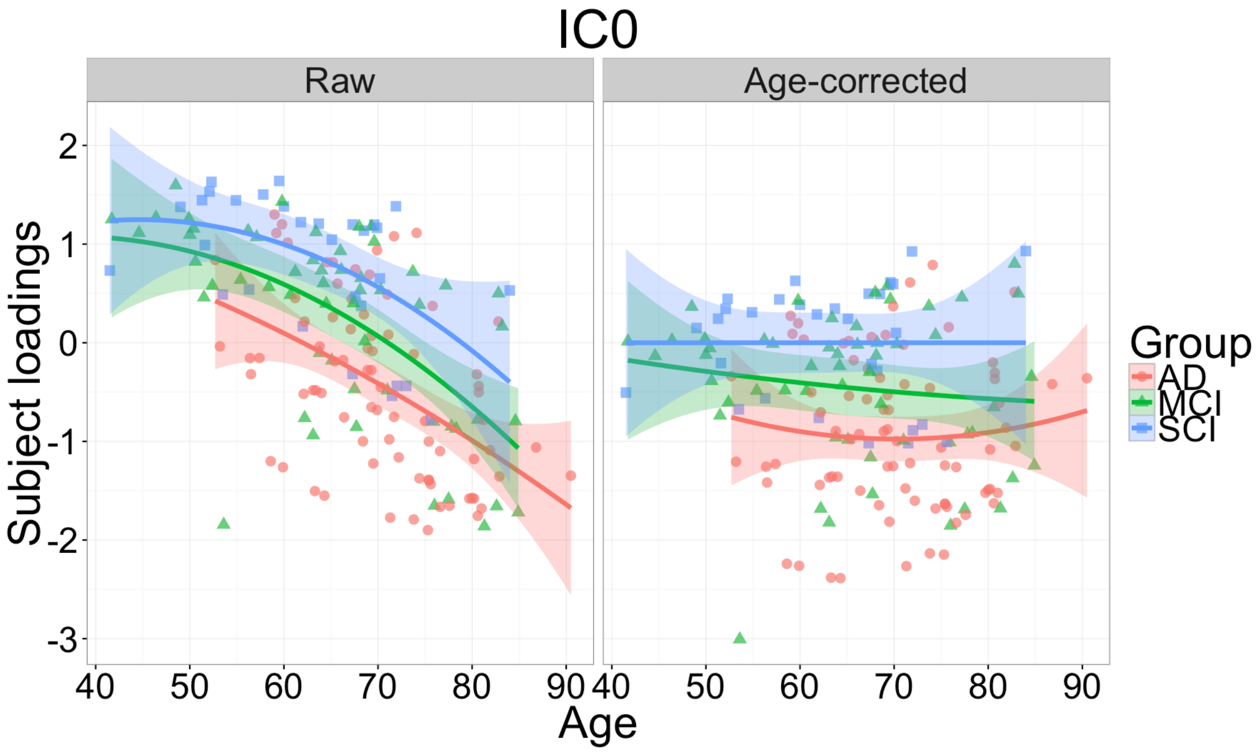

Figure S14. IC0: fitted linear regression line within each group of the raw and age-corrected subject loadings as a function of age. A linear regression with orthogonal second order polynomial was used to model the age effect within the SCI group and subsequently used to compute the residualized (age-corrected) subject loadings for subjects in all three groups.
